# Coupled In Silico Toxicology Models Reveal Equivalent Ecological Risks from BPA and Its Alternatives in Chinese Surface Waters

**DOI:** 10.3390/toxics13080671

**Published:** 2025-08-09

**Authors:** Jiawei Zhang, Jingzi Xiao, Huanyu Tao, Mengtao Zhang, Lu Lu, Changbo Qin

**Affiliations:** 1 Institute of Strategic Planning, Chinese Academy of Environmental Planning, Ministry of Ecology and Environment, Beijing 100041, China; zhangjw@caep.org.cn (J.Z.); xjzkkk0615@163.com (J.X.); 2 School of Environment, Tsinghua University, Beijing 100084, China; 3 College of Resource Environment and Tourism, Capital Normal University, Beijing 100048, China; 4 Department of Chemistry, University of California, Riverside, CA 92521, USA; huanyut@ucr.edu; 5 State Key Laboratory of Soil Pollution Control and Safety, Southern University of Science and Technology, Shenzhen 518055, China; zhangmt@sustech.edu.cn

**Keywords:** ecotoxicity, in silico models, bisphenols, risk quotients, Chinese surface waters

## Abstract

As bisphenol A (BPA) has gradually become restricted in production scenarios, the ecological risk level of its main replacement chemicals, i.e., bisphenol S (BPS) and bisphenol F (BPF), should be noted. To overcome the limitations of toxicity data, two kinds of in silico toxicology models (quantitative structure–activity relationship (QSAR) and interspecies correlation estimation (ICE) models) were used to predict enough toxicity data for multiple species. The accuracy of the coupled in silico toxicology models was verified by comparing experimental and predicted data results. Reliable predicted no-effect concentrations (PNECs) of 8.04, 35.2, and 34.2 μg/L were derived for BPA, BPS, and BPF, respectively, using species sensitivity distribution (SSD). Accordingly, the ecological risk quotient (RQ) values of BPA, BPS, and BPF for aquatic organisms were assessed in 32 major Chinese surface waters; they ranged from nearly 0 to 1.86, but were <0.1 in most cases, which indicated that the overall ecological risk level of BPA and its alternatives was low. However, in some cases, the ecological risks posed by BPA alternatives have reached equivalent levels to those posed by BPA (e.g., Liuxi River, Taihu Lake, and Pearl River), which requires further attention. This study provides evidence that the application of coupled in silico toxicology models can effectively predict toxicity data for new chemicals, avoiding time-consuming and laborious animal experiments. The main findings of this study can support environmental risk assessment and management for new chemicals that lack toxicity data.

## 1. Introduction

Bisphenol A (BPA) is a chemical with a high production volume and is widely used as an industrial raw material in polycarbonate plastic and epoxy resin for the production of plastic products and electronic equipment [1,2,3]. China consumes ∼3 million tonnes/year of BPA, and its in-use BPA stock is 14.0 million tonnes [4]. However, recent toxicological studies have shown the ecological and health toxicity effects of BPA at low concentrations, which warrant further BPA regulations and restrictions [5]. The United States, China, and the European Union have successively introduced strict regulations on BPA in materials in contact with food and in packaging containers [6,7]. BPA restrictions have stimulated the generation of replacement chemicals, including bisphenol S (BPS) and bisphenol F (BPF), for various applications [8,9]. BPS and BPF share close structural similarities with BPA (as shown in Table 1). These similarities make them ideal as replacements; however, because of these similarities, there also concerns that they may have the same ecological and health toxicity effects as BPA [10,11]. In their laboratory studies, Chen et al. [12] reported that many BPA analogs (including BPS and BPF) exhibit endocrine-disrupting effects, cytotoxicity, genotoxicity, reproductive toxicity, dioxin-like effects, and neurotoxicity. BPS and BPF have been shown to exhibit similar or even greater estrogenic and/or antiandrogenic activities compared to BPA [12,13]. Moreman, Lee, Trznadel, David, Kudoh and Tyler [10] conducted a comprehensive analysis on the toxicity and teratogenic effects of the bisphenols in zebrafish embryo larvae and found that the rank order for toxicity was BPA > BPF > BPS, while the rank order for estrogenicity was BPA = BPF > BPS. It was also reported that BPS and BPF exhibit similar antiandrogenic effects (they adversely affect basal testosterone secretion in human and mouse fetal testes at concentrations as low as 10 nmol/L) to BPA [14].

Due to safety concerns, the ecological risk of BPA and its replacement chemicals should be understood to determine whether they adhere to environmental regulations. The predicted no-effect concentration (PNEC) is the concentration of a chemical below which no adverse effects of exposure are measured in an ecosystem. A reliable PNEC can be calculated from the species sensitivity distribution (SSD) models with toxicity data for a diversity of species [15]. SSDs are cumulative probability distributions that are fitted to toxicity concentrations for different species, as described by Posthuma et al. [16]. The use of SSDs requires a lot of data (minimum sample sizes typically range from 5 to 10), which considerably limits the extent of its applicability [17]. Moreover, rather than short-term (acute) toxicity data, the acquisition of chronic data for a large number of species is recommended in SSD modeling. However, chronic toxicity data for BPA alternatives are still limited, which hinders the derivation of chronic PNECs. Moreover, increasing toxicity data through animal experiments is often impractical because experiments are time-consuming and laborious. Alternatively, many guidelines advocate for the development of experimental methods that do not involve animals for risk assessment, such as in silico toxicology models [18,19,20]. The term “in silico toxicology” generally refers to computational experiments, mathematical calculations, or scientific analyses of substances and the organization of substance-related data through computer-based analysis [21]. Leveraging molecular structure and properties, in silico toxicology models are often used in conjunction with in vitro and in vivo studies, providing a more comprehensive assessment of toxicity. While in silico models are increasingly used, their acceptance in regulatory frameworks is still evolving, with ongoing efforts underway to improve their transparency, their reproducibility, and confidence in their predictions [22].

The use of in silico toxicology models to extrapolate and predict toxicity data during chemical risk assessment is applied internationally [23,24,25,26]. Quantitative structure–activity relationship (QSAR) and interspecies correlation estimation (ICE) models are two commonly used techniques among the in silico approaches [25]. QSAR is a regression or classification model that can be used to predict toxicity data of chemicals based on the knowledge of their chemical structure [27]. QSAR models are available for free or as commercial software. For example, the VEGA platform (https://www.vegahub.eu/ (accessed on 20 December 2024)) is a free JAVA technology-based software that provides tens of QSAR models to predict the toxicity properties of chemicals [28]. VEGA is widely accepted by international scientific and industrial communities. For example, it has been used by ECHA to identify substances suspected to meet the REACH Annex III criteria [29]. For instance, in a recent study, Vračko and Lagares [30] addressed the in silico toxicity of BPA alternatives with VEGA QSAR models for *D. magna*, *P. promelas*, and *O. latipes*. An ICE model is a more recent statistical extrapolation method that uses available toxicity data from surrogate species to predict the toxicity of untested species, which may prove useful for the development of SSD models to derive reliable PNECs [31]. ICE models were first developed by the USEPA and have become globally accepted due to their continuous development. Currently, the Web-ICE application (https://www.epa.gov/webice/ (accessed on 26 December 2024)) provides interspecies extrapolation models for toxicity via a user-friendly Internet platform, which can be widely used for ecological risk assessment [31,32]. For example, Tao et al. [33] used selected ICE models from the Web-ICE application combined with SSD models they constructed to assess the ecological risk of the plasticizer dibutyl phthalate (DBP) and alternative di-isobutyl phthalate (DiBP) in the surface waters.

Independent QSAR and ICE models have advantages and disadvantages [25]. While QSAR models can provide toxicity data for certain species, SSD modeling for ecological risk assessment requires toxicity data for a broad diversity of species. In an ICE model, if toxicity data are available for surrogate species, toxicity to the predicted taxon can be estimated for a particular interspecies pair. Hence, researchers in the field of ecological risk assessment realized that these two types of in silico models could be coupled [25,34]. QSAR can estimate toxicity based on structure, filling data gaps where experimental data is unavailable. Meanwhile, ICE models extrapolate the toxicity of a chemical in one species to a wider range of species, reducing the need for extensive animal testing. The generated toxicity data can be used to construct SSDs, which are crucial for deriving PNECs. In our previous study [35], we used coupled QSAR-ICE-SSD models to extrapolate the toxicity of per- and polyfluoroalkyl substances (PFASs) and then compared the PNEC results derived from actual toxicity data with the model-based results. We found that the coupled models had a certain degree of accuracy and can be used as an alternative method in the screening of ecological risk assessment. Several studies have also demonstrated the effectiveness of this approach [23,25,32,36,37,38].

In this study, we aim to (1) develop coupled in silico toxicology models, i.e., QSAR and ICE models, to obtain enough toxicity data for BPA and its replacement chemicals; (2) validate the coupled in silico toxicology models by comparing the results of the measured data and predicted data; and (3) collect the environmental concentrations and calculate the ecological risk levels of BPA, BPS, and BPF in the Chinese surface waters.

## 2. Materials and Methods

### 2.1. Selection of Toxicity Data and Environmental Concentrations

Data on BPA, BPS, and BPF chronic toxicity in freshwater aquatic organisms were collected from the USEPA ECOTOX Database (https://cfpub.epa.gov/ecotox/ (accessed on 1 December 2024)) as well as published papers from Web of Science. Then, the collected data were screened according to the following rules: the endpoints were no-observed-effect concentrations (NOECs); the effects were associated with chronic lethal toxicity; the duration of exposure must be at least ≥ 4 days for algae and ≥21 days for other species; the test method must conform to the standard test procedure recommended by the national standards of China [39,40].

The environmental concentrations of BPA, BPS, and BPF in Chinese surface waters primarily came from papers published in Web of Science. Sampling, sample preparation, and instrumental analysis methods were required to have appropriate quality control and quality assurance measures or comply with relevant guidelines. Moreover, the statistical characteristics (e.g., ranges, or mean values) of environmental concentration at each location needed to be provided.

### 2.2. Development of Coupled In Silico Toxicology Models

To enhance the toxicity data for PNEC calculations, two in silico toxicology models (i.e., QSAR and ICE) were combined. By providing a broader and more comprehensive toxicity dataset, the combination of in silico models reduces the need for overly conservative assessment factors, leading to more accurate PNEC values. First, QSAR models were used to obtain predicted toxicity data for representative aquatic organisms of three different trophic levels. Then, ICE models were used to extrapolate the predicted toxicity values of more organisms based on the QSAR toxicity values. Finally, sufficient toxicity data from these coupled in silico toxicology models could be obtained and used for further SSD modeling.

Three available QSAR models in the VEGA platform related to aquatic chronic toxicity in fish (medaka, *Oryzias latipes*), crustaceans (water flea, *Daphnia magna*), and algae (*Pseudokirchneriella subcapitata*) were selected. The models used experimental data with a tree ensemble random forest algorithm. More detailed technical guidelines can be found on the VEGA website (https://www.vegahub.eu/ (accessed on 20 December 2024)).

Six ICE models in the US EPA Web-ICE database were selected according to the following guidelines (as shown in Table 2): coefficient of determination (R^2^) > 0.6; mean square error (MSE) < 0.95; slope > 0.6; cross-validation success rate > 60%; *p*-value < 0.01; narrow confidence intervals [32,41]. More detailed technical guidelines can be found on the Web-ICE website (https://www3.epa.gov/webice/ (accessed on 26 December 2024)).

Although variations exist in some of the technical details and associated software tools employed, the fundamental SSD approach employed by jurisdictions around the world remains similar [42,43,44]. Log-logistic, log-normal, and Burr III type (including Burr III, ReWeibull, and Weibull) distributions are commonly used methods for SSD construction [15,45]. In this study, the SSD models were implemented in R version 4.1 with the R package “ssdtools” (version 2.3.0) [46]. The “ssdtools” package uses Maximum Likelihood to fit distributions such as the log-normal, log-logistic, log-Gumbel (also known as the inverse Weibull) distributions, in which confidence intervals on hazard concentrations and proportions are produced by bootstrapping. In this study, to fit the SSD curves, the Anderson–Darling test, Kolmogorov–Smirnov test, and Akaike’s information criterion (AIC) were applied to assess the goodness of fit. The lower the AIC value, the better the fit of the models [47]. As shown in Table S1, compared with the other distributions, in most cases, the log-normal-distribution-based SSD models had a good curve-fitting result when using AIC as a measure of the relative quality of fit. Therefore, the SSD models were developed using the log-normal distribution. The log-normal distribution is a right-skewed continuous probability distribution that is used for modeling various natural phenomena. The probability density function (Equation (1)) for the log-normal distribution is defined by the two parameters *μ* and *σ*, where *x* > 0:

(1) $$f\left(x\right)=\frac{1}{x\sigma \sqrt{2\pi }}e^{-\frac{1}{2}({\frac{\ln\left(x\right)-\mu }{\sigma })}^{2}}$$

where *μ* is the location parameter, and σ the scale parameter of the distribution; when log-normal data is transformed using logarithms, *μ* can then be viewed as the mean (of the transformed data) and σ as the standard deviation (of the transformed data).

### 2.3. Calculation of HC5s and PNECs

According to relevant guidelines, the hazardous concentration for 5% of species (HC5) and its 95% confidence interval (CI) can be calculated from the SSD models as a predicted quantile value of 5% of the curve. HC5 was used to obtain the PNEC using Equation (2).

(2) $$PNEC=\frac{HC5}{AF}$$

where AF is the assessment factor, which has a value between 1 and 5, reflecting the uncertainty of the data, and may make PNEC more conservative.

### 2.4. Ecological Risk Assessment

The ecological risk of BPA, BPS, and BPF to aquatic organisms in Chinese surface waters was assessed using the risk quotient (RQ) method. An RQ is the ratio of a point estimate of environmental exposure concentration and a point estimate of toxicity endpoints for the target chemical, as shown in Equation (3). According to the calculated RQs, the ecological risk was characterized into three levels: high risk (RQ ≥ 1), moderate risk (0.1 ≤ RQ ≤ 1), and low risk (0.01 ≤ RQ < 0.1) [40,48].

(3) $$RQ=\frac{MEC}{PNEC}$$

where MEC is the measured environmental concentration of the target chemical.

## 3. Results and Discussion

### 3.1. Occurrence of BPA, BPS, and BPF in Chinese Surface Waters

Table 3 shows the concentrations of BPA, BPS, and BPF in 32 major Chinese surface waters, including the min–max and mean values. The mean BPA concentration ranges in all samples were 8.38–922 ng/L. The maximum BPA concentration reached 7480 ng/L. The five highest concentrations of BPA were 75.6–7480 ng/L (Liuxi River), 118–1770 ng/L (Zhujiang River), 23.7–2180 ng/L (Dongjiang River), 19–702 ng/L (River, Port, Lake and Chanel of Jiangyan District), and 85.9–586.4 ng/L (Yangtze River and Urban River in Nanjing). In the waters of Liuxi River, Taihu Lake and Pearl River, the BPS and BPF concentrations were even higher than the BPA concentrations, as indicated in Table 3. The mean BPS concentration range of all samples was 0.34–3720 ng/L. The five highest concentrations of BPS were 19.9–65,600 ng/L (Liuxi River), 0–135 ng/L (Pearl River), 4.5–1600 ng/L (Taihu Lake), 4.5–1569 ng/L (Taihu Lake), and 6.56–293 ng/L (Taihu Lake, Gehu Lake and Rivers). The mean BPF concentration range of all samples was 0.016–773 ng/L. The five highest concentrations of BPF were 448–1110 ng/L (Pearl River), 200–220 ng/L (Fangting River), 130–220 ng/L (Bulao River), 110–230 ng/L (Zhongyun River), and 110–220 ng/L (Yi River).

Yamazaki et al. [49] reported that BPA concentrations were in the range of several tens to several hundreds of nanograms per liter in most of the rivers they surveyed, and the concentrations of BPF were highest among the BPA substitutes, suggesting that BPF might account for the majority of BPA substitutes on the market. BPF concentrations in surface water samples collected from Japan, Korea, and China were 1–2 orders of magnitude higher than those of BPA, which suggested that BPF is the major bisphenol contaminant in the surface waters of several Southeast Asian countries [49]. In this study, based on publicly available data, the environmental concentrations of BPA and its major alternatives (i.e., BPS and BPF) in Chinese surface waters were obtained and analyzed. The results showed that in most cases, the concentrations of BPA still exceeded those of its alternatives (Table 3). However, in certain surface waters, the concentrations of alternatives have far surpassed those of BPA, indicating that the potential environmental pollution caused by these alternatives requires further attention.

**Table 3:** Occurrence of BPA, BPS, and BPF in Chinese surface waters.

No.	Location	Sampling Year	Pre-Treatment and Detection Method	Concentration (Range with Mean Value, ng/L)			Reference
				BPA	BPS	BPF	
1	Luoma Lake	2015	SPE + HPLC-MS/MS	49–110 (86)	0–94 (21)	3.5–14 (6.8)	[50]
2	Luoma Lake	2020	SPE + UPLC-MS/MS	120–280 (200)	3.2–7.7 (5.45)	87.4–230 (159)	[51]
3	Taihu Lake	2013	SPE + UPLC-MS/MS	4.2–14 (8.5)	0.28–67 (6)	0–5.6 (0.83)	[52]
4	Taihu Lake	2015	SPE + HPLC-MS/MS	27–565 (86)	4.5–1569 (101)	0–1634 (114)	[53]
5	Taihu Lake	2016	SPE + HPLC-MS/MS	28–560 (97)	4.5–1600 (120)	0–1600 (140)	[50]
6	Taihu Lake	2016	SPE + HPLC-MS/MS	19–68 (26)	4.1–160 (16)	26–720 (78)	[54]
7	Taihu Lake, Gehu Lake and Rivers	2018	SPE + LC-MS/MS	47.8–633 (196)	6.56–293 (56.1)	0.48–36.7 (5.82)	[55]
8	Bulao River	2020	SPE + UPLC-MS/MS	220–310 (265)	5.5–7.8 (6.65)	130–220 (175)	[51]
9	Dongjiang River	2015	SPE + UPLC-MS/MS	23.7–2180 (406)	0.07–133 (12.7)	0.98–255 (25.2)	[2]
10	Fangting River	2020	SPE + UPLC-MS/MS	250–290 (270)	3.6–6.1 (4.85)	200–220 (210)	[51]
11	Guangzhou Section of Pearl River	2022	SPE + UPLC-MS/MS	60.5–187.5 (124)	1.7–102.1 (51.9)	5.4–118.8 (62.1)	[56]
12	Hunhe river	2013	SPE + UPLC-MS/MS	4.4–107 (40)	0.61–46 (11)	ND	[52]
13	Irrigation Rivers in Zhangjiagang City	2023	SPE + UPLC-MS/MS	4.66–64.77 (22.19)	0–74.04 (6.42)	0–22.88 (1.04)	[57]
14	Lanzhou Section of Yellow River	2017	SPE + HPLC-MS/MS	7.8–138.5 (42.6)	0–19.4 (5.6)	/	[58]
15	Laoyi River	2020	SPE + UPLC-MS/MS	210–220 (215)	4.2–4.7 (4.45)	91.9–130 (111)	[51]
16	Liaohe river	2013	SPE + UPLC-MS/MS	5.9–141 (47)	0.22–52 (14)	ND	[52]
17	Liuxi River	2016	LLE/SPE + HPLC-MS/MS	75.6–7480 (922)	19.9–65,600 (3720)	0–474 (82.8)	[59]
18	Luoma Lake Inflow Rivers	2020	SPE + UPLC-MS/MS	120–310 (215)	3.6–7.8 (5.7)	91.9–230 (161)	[60]
19	Pearl River	2015	SPE + LC-MS/MS	0–98 (73)	0–135 (135)	448–1110 (773)	[49]
20	River, Port, Lake and Chanel of Jiangyan District	2018	SPE + UPLC-MS/MS	19–702 (371.5)	3.4–83.5 (37.1)	0–270.6 (42.9)	[61]
21	Rivers, Lakes and Reservoirs	2017	SPE + UPLC-MS/MS	0–34.9 (12.8)	0–5.2 (1.1)	0–12.56 (2.18)	[62]
22	West River	2015	SPE + LC-MS/MS	0–43 (43)	ND	0–105 (64)	[49]
23	Yangtze River and Urban River in Nanjing	2018	SPE + UPLC-MS/MS	85.9–586.4 (315.8)	12.9–143.4 (51.6)	1.4–27.3 (12.2)	[63]
24	Yangtze River and Urban River in Nanjing	2018	SPE + UPLC-MS/MS	120–554 (253)	2.24–73.3 (39.2)	0–4.76 (2.2)	[64]
25	Yi River	2020	SPE + UPLC-MS/MS	120–170 (145)	4.1–6.4 (5.25)	110–220 (165)	[51]
26	Zhongyun River	2020	SPE + UPLC-MS/MS	180–300 (240)	4.2–6 (5.1)	110–230 (170)	[51]
27	Zhujiang River	2015	SPE + UPLC-MS/MS	118–1770 (471)	16.6–103 (44.5)	6.54–34.4 (12.2)	[2]
28	Pearl River Delta	2020	SPE + HPLC-MS/MS	1.7–93 (9.5)	0.039–7 (0.54)	0–1.6 (0.016)	[65]
29	Pearl River Estuary	2017	SPE + UPLC- Q-Exactive Orbitrap MS	9.48–173 (24.6)	1.6–59.8 (10.3)	2.37–282 (35)	[66]
30	Seawater of Beibu Gulf	2017	SPE + UPLC-MS/MS	5.26–12.04 (8.38)	0.07–0.63 (0.34)	ND	[67]
31	Seawater of East China Sea	2019	SPE + UPLC-MS/MS	2.7–52 (23)	0.15–12 (2.2)	ND	[68]
32	Seawater of Hangzhou bay	2012	SPE + UPLC-MS/MS	6.59–74.58 (26)	0.29–18.99 (4.6)	0–3.47 (3.2)	[69]

Note: ND means not detected; / means no data; SPE means solid phase extraction; LLE means liquid–liquid extraction; HPLC means high-performance liquid chromatography; UPLC means ultra-performance liquid chromatography; MS/MS means tandem mass spectrometry.

### 3.2. Validation of Coupled In Silico Toxicology Models and Calculation of PNECs

**Figure 1:**
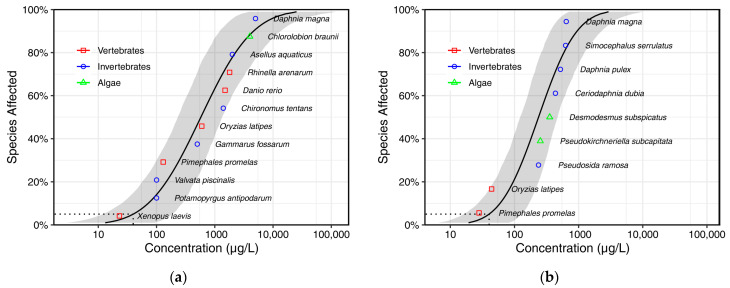
SSD models of chronic lethal toxicity data for BPA ((**a**). using experimental toxicity data; (**b**). using predicted toxicity data from the coupled in silico toxicology models).

Table 4 shows chronic lethal BPA toxicity data for twelve species, including five vertebrates, six invertebrates, and one alga. The toxicity data ranged from 23 μg/L (*Xenopus laevis*) to 5000 μg/L (*Daphnia magna*). Using experimental toxicity data, the SSD model for BPA was developed, as shown in Figure 1a. Based on the coupled in silico toxicology models mentioned in Section 2.2 (i.e., three available QSAR models on the VEGA platform and six ICE models in the US EPA Web-ICE database), toxicity data for nine species, including two vertebrates, five invertebrates, and two algae, were obtained. Using these predicted toxicity data from the coupled in silico toxicology models, the SSD model for BPA was developed, as shown in Figure 1b.

According to the results of the goodness-of-fit tests of the SSD models, calculated with the R package “ssdtools”, as described in Section 2.2, log-normal SSD models had the best goodness-of-fit results (as shown in Table S1). The sample size for constructing SSD models met the relevant requirements, indicating that the SSD models had high robustness and accuracy.

The accuracy of the coupled in silico toxicology models was verified. As indicated in Table 5, the HC5 values from the SSD models using experimental toxicity data and predicted toxicity data from the coupled in silico toxicology models were calculated to be 39.8 (95% CI 12.1–186) μg/L and 40.2 (95% CI 16.2–129) μg/L, respectively. The difference between these values was not significant, indicating that the prediction method from the coupled in silico toxicology models is accurate and effective for BPA.

The combination of QSAR and ICE models could enable researchers to leverage the advantages of their prediction abilities, making this an increasingly popular topic in the scientific community [25]. QSAR-based toxicity data can be used as the input into the ICE models to generate a set of toxicity data for diverse species, and then used in SSD models. Several studies have demonstrated the comparability of HC values derived from this kind of coupled in silico toxicology model to those from SSD models based on measured toxicity data. In the very beginning, Barron, Jackson and Awkerman [34] from the USEPA first assessed whether SSD models could be generated with reasonable accuracy using only coupled in silico toxicology modeling of toxicity to aquatic organisms. Each QSAR-based toxicity dataset was used as an input to Web-ICE to generate estimated in silico HC5 values. He, Tang, Zhao, Fan, Dyer, Belanger and Wu [25] proposed a conceptual framework indicating that this coupled in silico toxicology modeling method may be suitable for developing predicted water quality benchmarks. More recently, combined QSAR-ICE models were used in the calculation of PNECs for polyfluoroalkyl substances, linear alkylbenzene sulfonate, and alkylphenol substances and showed relatively accurate prediction results [23,35,38].

**Figure 2:**
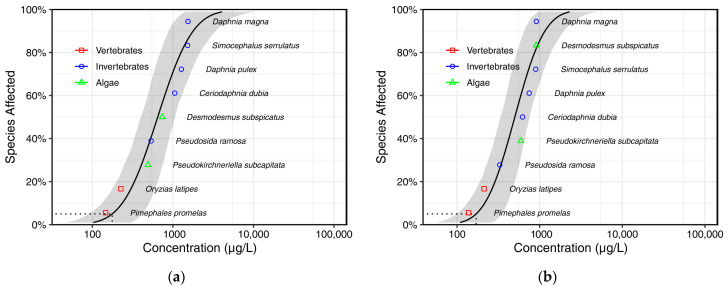
SSD models of chronic lethal toxicity data from the coupled in silico toxicology models ((**a**). BPS; (**b**). BPF).

Based on the above, in this study, the SSD models for BPS and BPF using predicted toxicity data from the coupled in silico toxicology models were developed, as shown in Figure 2. This makes it possible to derive PNECs when an SSD model cannot be directly constructed due to a lack of experimental toxicity data. Again, the log-normal SSD models showed satisfactory goodness-of-fit results (Table S1). Accordingly, the HC5 values for BPS and BPF were calculated to be 176 (95% CI 90.5–415) μg/L and 171 (95% CI 98.1–347) μg/L, respectively (Table 5). The HC5 values for BPS and BPF were higher than that for BPA, albeit within one order of magnitude, indicating a likely environmental hazard to aquatic organisms. From a safety perspective, the potential hazards of BPA replacement chemicals with the same concentration are similar to those of BPA, indicating that this replacement strategy warrants further consideration.

According to Equation (2), the AF was set to 5 in this study [80], and then PNEC values for BPA, BPS, and BPF were calculated to be 7.96 μg/L, 35.2 μg/L, and 34.2 μg/L, respectively. These PNEC values were used in the ecological risk assessment, which is discussed in the following section.

### 3.3. Ecological Risk of BPA, BPS, and BPF in Chinese Surface Waters

**Figure 3:**
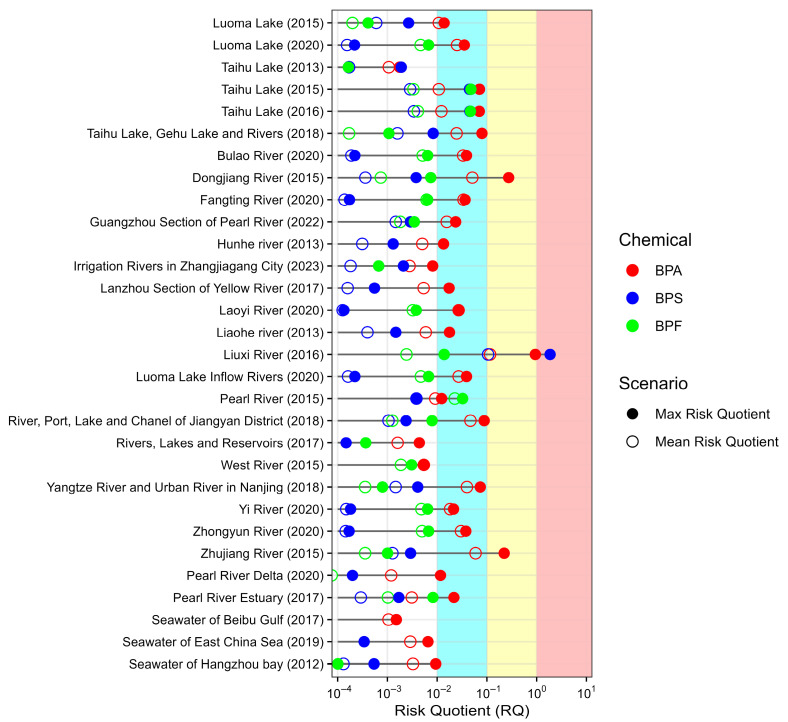
RQs of BPA, BPS, and BPF in Chinese surface waters (the hollow circles represent the RQs calculated using the mean concentration value, and the solid circles represent the RQs calculated using the maximum concentration value; the different colors, i.e., blue, yellow, red, in the background represent different ecological risk levels, i.e., low risk (0.01 ≤ RQ < 0.1), moderate risk (0.1 ≤ RQ ≤ 1), and high risk (RQ ≥ 1)).

The ecological risk of BPA, BPS, and BPF to aquatic organisms in 32 major Chinese surface waters was assessed using the RQ method, as illustrated in Figure 3. The mean RQ values (representing the average case) were 0.00105–0.116, 0.00001–0.106, and 0–0.0226 for BPA, BPS, and BPF, respectively. The max RQ values (representing the most severe case) reached 0.94, 1.86, and 0.0478 for BPA, BPS, and BPF, respectively. Overall, the risk level (29 in 32 cases) remained low (i.e., RQ values < 0.1). It should be noted that in the Liuxi River, Taihu Lake, and Pearl River samples, the risk level of BPS/BPF was equal to or even greater than that of BPA.

Despite using the SSD method to derive PNECs for ecological risk assessment, uncertainty was unavoidable. The uncertainty in this study mainly came from (1) the uneven spatiotemporal distribution of chemicals in the water environment; (2) the low ecological relevance of toxicity data generated under laboratory conditions; and (3) methodological errors from the construction of the SSD model. Because of this uncertainty, the risk assessment results may differ significantly from real life. For example, the fitting results of SSD models may not be sufficient to simulate the situation in a real ecosystem. According to the literature, compared to real situations, the ecological risks predicted in the research are often overestimated [81]. Therefore, the risk assessment results of this study can only guide risk-based decision-making, and further research is needed.

### 3.4. Implications and Limitations

In this study, coupled in silico toxicology models including QSAR and ICE were developed and applied in the ecological risk assessment of BPA and its replacement chemicals. The accuracy of these coupled in silico toxicology models was verified by comparing their predicted and measured HC5 values.

For most new chemicals, especially replacement chemicals, toxicity data was very limited. Traditionally, supplementing toxicity data requires animal experiments. However, these are time-consuming and often considered unethical. As a result, in recent years, computational methods have been advocated for by the scientific community and relevant risk assessment guidelines. In this study, the application of coupled in silico toxicology models proved to be an effective method to augment toxicity data for the construction of SSD models.

There were several major limitations to the coupled in silico toxicology models constructed in this study. First, the model was only validated by one chemical, limited by the lack of toxicity data. Second, it did not include other important toxicity endpoints, such as developmental toxicity, reproductive toxicity, etc. Finally, in risk assessments, local species should be considered where possible to derive PNEC values. However, due to the lack of toxicity data, this study did not consider the local species composition.

## 4. Conclusions

The concentrations of BPA, BPS, and BPF in 32 major Chinese surface waters were determined, with maximum values of 7480 ng/L, 65,600 ng/L, and 1634 ng/L, respectively. Coupled in silico QSAR and ICE toxicology models were developed and verified by comparing their predicted and measured HC5 values. The PNEC values for BPA, BPS, and BPF were calculated to be 7.96 μg/L, 35.2 μg/L, and 34.2 μg/L, respectively. The RQ results showed that the overall risk level remained low (i.e., RQ values < 0.1); however, in some cases, BPA alternatives showed similar ecological risks to BPA (e.g., Liuxi River, Taihu Lake, and Pearl River). The coupled in silico models effectively augmented toxicity data to enable ecological risk assessment for BPA and its replacements, despite the limitations of being validated on only one chemical, not addressing key endpoints like developmental/reproductive toxicity, and the lack of consideration of local species.

## Figures and Tables

**Table 1:** Physicochemical properties for BPA, BPS, and BPF.

Chemical	Abbr.	Structure	CAS Number	Molecular Formula	Molecular Weight (g/mol)	Solubility in Water (mg/L)	Log K_ow_	Log K_oc_	Half-Life in Water (days)	BCF
Bisphenol A	BPA	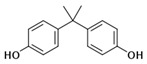	80-05-7	C_15_H_16_O_2_	228.29	300	3.41	4.88	37.5	71.9
Bisphenol S	BPS	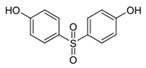	80-09-1	C_12_H_10_O_4_S	250.27	1100	1.65	2.5	37.5	3.16
Bisphenol F	BPF	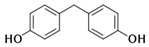	620-92-8	C_13_H_12_O_2_	200.24	540	2.91	4.47	15	34.7

Note: K_ow_ means octanol–water partition coefficient; K_oc_ means soil adsorption coefficient; BCF means bioconcentration factor; All physicochemical properties were sourced from commonly used publicly accessible chemical databases (accessed on 12 May 2025) including the EPA CompTox Chemicals Dashboard (https://comptox.epa.gov/dashboard/), PubChem database (https://pubchemdocs.ncbi.nlm.nih.gov/), and ChemSpider database (http://www.chemspider.com/).

**Table 2:** Selected ICE models from the USEPA Web-ICE database.

Predicted Species	Surrogate Species	R^2^	*p*-Value	MSE	Cross-Validation Success (%)	Slope	Intercept
*Pimephales promelas*	*Oryzias latipes*	0.92	<0.001	0.26	78	1.01	−0.21
*Ceriodaphnia dubia*	*Daphnia magna*	0.95	<0.001	0.26	81	1	−0.19
*Daphnia pulex*	*Daphnia magna*	0.97	<0.001	0.12	90	1.01	−0.14
*Simocephalus serrulatus*	*Daphnia magna*	0.88	<0.001	0.21	87	1	−0.03
*Pseudosida ramosa*	*Daphnia magna*	0.87	0.006	0.57	67	0.93	−0.24
*Desmodesmus subspicatus*	*Pseudokirchneriella subcapitata*	0.96	<0.001	0.31	84	1.1	−0.11

Note: R^2^ means coefficient of determination; *p*-value means the probability that a particular statistical measure; MSE means mean squared error.

**Table 4:** Selected chronic lethal toxicity data for BPA.

No.	Species	Group	Concentration (μg/L)	Observed Duration (days)	Reference
1.	*Chlorolobion braunii*	Algae	3995	4	[70]
2.	*Asellus aquaticus*	Crustaceans	2000	21	[71]
3.	*Daphnia magna*	Crustaceans	5000	21	[8]
4.	*Gammarus fossarum*	Crustaceans	500	103	[72]
5.	*Chironomus tentans*	Insects	1400	4	[73]
6.	*Potamopyrgus antipodarum*	Molluscs	100	28	[74]
7.	*Valvata piscinalis*	Molluscs	100	28	[74]
8.	*Rhinella arenarum*	Amphibians	1799	14	[75]
9.	*Xenopus laevis*	Amphibians	23	84	[76]
10	*Danio rerio*	Fish	1500	21	[77]
11.	*Oryzias latipes*	Fish	598	44	[78]
12.	*Pimephales promelas*	Fish	130	164	[79]

**Table 5:** Calculation of PNECs for BPA, BPS, and BPF.

Chemical	Dataset of SSD	HC5 and Its 95% CI (μg/L)	Assessment Factor	PNEC (μg/L)
BPA	Experimental toxicity data	39.8 (12.1–186)	5	7.96
BPA	Predicted toxicity data from the coupled in silico toxicology models	40.2 (16.2–129)	5	8.04
BPS	Predicted toxicity data from the coupled in silico toxicology models	176 (90.5–415)	5	35.2
BPF	Predicted toxicity data from the coupled in silico toxicology models	171 (98.1–347)	5	34.2

Note: HC5 means the hazardous concentration for 5% of species; PNEC means the predicted no-effect concentration.

## Data Availability

The data that support the findings of this study are available from the corresponding author, upon reasonable request.

## Supplementary Materials

The following supporting information can be downloaded at https://www.mdpi.com/article/10.3390/toxics13080671/s1, Table S1: The results of the goodness-of-fit tests of SSD models; Table S2: RQ values of BPA, BPS, and BPF in Chinese surface waters.
