# Supplementary material for: Coupled In Silico Toxicology Models Reveal Equivalent Ecological Risks from BPA and Its Alternatives in Chinese Surface Waters
Source: Toxics. 2025 Aug 9;13(8):671. doi: 10.3390/toxics13080671 (PMC12390238; doi:10.3390/toxics13080671)
Supplement: Supplementary file 1 [file toxics-13-00671-s001.zip › toxics-3760605-supplementary.pdf]

Support Information

Coupled *in silico* Toxicology Models Reveal Equivalent Ecological Risks from BPA and its Alternatives in Chinese Surface Waters

Table S1. The results of the goodness-of-fit tests of SSD models.

Compound	SSD Distribution	Anderson-Darling Statistic	Kolmogorov-Smirnov Statistic	Akaike's Information Criterion
BPA (using experimental toxicity data)	log-Gumbel	0.576706	0.204265	204.9197
	log-logis	0.435351	0.186906	203.1613
	log-norm	0.449564	0.207369	202.2864
BPA (using predicted toxicity data)	log-Gumbel	0.971574	0.319594	132.5253
	log-logis	0.701147	0.205981	129.3932
	log-norm	0.815842	0.277337	129.3608
BPS	log-Gumbel	0.519832	0.207654	144.1333
	log-logis	0.346455	0.155404	142.3888
	log-norm	0.389598	0.175058	141.8549
BPF	log-Gumbel	0.746875	0.287377	136.5577
	log-logis	0.607454	0.217959	134.2608
	log-norm	0.669114	0.269003	133.836

Table S2. Risk quotient (RQ) values of BPA, BPS, and BPF in Chinese surface waters.

Location (Sampling Year)	Compound	Mean RQ	Max RQ
Bulao River (2020)	BPA	0.033291	0.038945
Bulao River (2020)	BPF	0.005117	0.006433
Bulao River (2020)	BPS	0.000189	0.000222
Dongjiang River (2015)	BPA	0.051005	0.273869
Dongjiang River (2015)	BPF	0.000737	0.007456
Dongjiang River (2015)	BPS	0.000361	0.003778
Fangting River (2020)	BPA	0.03392	0.036432
Fangting River (2020)	BPF	0.00614	0.006433
Fangting River (2020)	BPS	0.000138	0.000173
Guangzhou Section of Pearl River (2022)	BPA	0.015578	0.023555
Guangzhou Section of Pearl River (2022)	BPF	0.001816	0.003474
Guangzhou Section of Pearl River (2022)	BPS	0.001474	0.002901
Hunhe River (2013)	BPA	0.005025	0.013442
Hunhe River (2013)	BPS	0.000313	0.001307
Irrigation Rivers in Zhangjiagang City (2023)	BPA	0.002788	0.008137
Irrigation Rivers in Zhangjiagang City (2023)	BPF	0.00003	0.000669
Irrigation Rivers in Zhangjiagang City (2023)	BPS	0.000182	0.002103
Lanzhou Section of Yellow River (2017)	BPA	0.005352	0.017399
Lanzhou Section of Yellow River (2017)	BPS	0.000159	0.000551
Laoyi River (2020)	BPA	0.02701	0.027638
Laoyi River (2020)	BPF	0.003246	0.003801

Location (Sampling Year)	Compound	Mean RQ	Max RQ
Laoyi River (2020)	BPS	0.000126	0.000134
Liaohe River (2013)	BPA	0.005905	0.017714
Liaohe River (2013)	BPS	0.000398	0.001477
Liuxi River (2016)	BPA	0.115829	0.939698
Liuxi River (2016)	BPF	0.002421	0.01386
Liuxi River (2016)	BPS	0.105682	1.863636
Luoma Lake (2015)	BPA	0.010804	0.013819
Luoma Lake (2015)	BPF	0.000199	0.000409
Luoma Lake (2015)	BPS	0.000597	0.00267
Luoma Lake (2020)	BPA	0.025126	0.035176
Luoma Lake (2020)	BPF	0.004649	0.006725
Luoma Lake (2020)	BPS	0.000155	0.000219
Luoma Lake Inflow Rivers (2020)	BPA	0.02701	0.038945
Luoma Lake Inflow Rivers (2020)	BPF	0.004708	0.006725
Luoma Lake Inflow Rivers (2020)	BPS	0.000162	0.000222
Pearl River (2015)	BPA	0.009171	0.012312
Pearl River (2015)	BPF	0.022602	0.032456
Pearl River (2015)	BPS	0.003835	0.003835
Pearl River Delta (2020)	BPA	0.001193	0.011683
Pearl River Delta (2020)	BPF	/	0.000047
Pearl River Delta (2020)	BPS	0.000015	0.000199
Pearl River Estuary (2017)	BPA	0.00309	0.021734

Location (Sampling Year)	Compound	Mean RQ	Max RQ
Pearl River Estuary (2017)	BPF	0.001023	0.008246
Pearl River Estuary (2017)	BPS	0.000293	0.001699
River, Port, Lake and Chanel of Jiangyan District (2018)	BPA	0.046671	0.088191
River, Port, Lake and Chanel of Jiangyan District (2018)	BPF	0.001254	0.007912
River, Port, Lake and Chanel of Jiangyan District (2018)	BPS	0.001054	0.002372
Rivers, Lakes and Reservoirs (2017)	BPA	0.001608	0.004384
Rivers, Lakes and Reservoirs (2017)	BPF	0.000064	0.000367
Rivers, Lakes and Reservoirs (2017)	BPS	0.000031	0.000148
Seawater of Beibu Gulf (2017)	BPA	0.001053	0.001513
Seawater of Beibu Gulf (2017)	BPS	0.00001	0.000018
Seawater of East China Sea (2019)	BPA	0.002889	0.006533
Seawater of East China Sea (2019)	BPS	0.000063	0.000341
Seawater of Hangzhou Bay (2012)	BPA	0.003266	0.009369
Seawater of Hangzhou Bay (2012)	BPF	0.000094	0.000101
Seawater of Hangzhou Bay (2012)	BPS	0.000131	0.000539
Taihu Lake (2013)	BPA	0.001068	0.001759
Taihu Lake (2013)	BPF	0.000024	0.000164
Taihu Lake (2013)	BPS	0.00017	0.001903
Taihu Lake (2015)	BPA	0.010804	0.07098
Taihu Lake (2015)	BPF	0.003333	0.047778
Taihu Lake (2015)	BPS	0.002869	0.044574

Location (Sampling Year)	Compound	Mean RQ	Max RQ
Taihu Lake (2016)	BPA	0.012186	0.070352
Taihu Lake (2016)	BPF	0.004094	0.046784
Taihu Lake (2016)	BPS	0.003409	0.045455
Taihu Lake, Gehu Lake and Rivers (2018)	BPA	0.024623	0.079523
Taihu Lake, Gehu Lake and Rivers (2018)	BPF	0.00017	0.001073
Taihu Lake, Gehu Lake and Rivers (2018)	BPS	0.001594	0.008324
West River (2015)	BPA	0.005402	0.005402
West River (2015)	BPF	0.001871	0.00307
Yangtze River and Urban River in Nanjing (2018)	BPA	0.039673	0.073668
Yangtze River and Urban River in Nanjing (2018)	BPF	0.000357	0.000798
Yangtze River and Urban River in Nanjing (2018)	BPS	0.001466	0.004074
Yi River (2020)	BPA	0.018216	0.021357
Yi River (2020)	BPF	0.004825	0.006433
Yi River (2020)	BPS	0.000149	0.000182
Zhongyun River (2020)	BPA	0.030151	0.037688
Zhongyun River (2020)	BPF	0.004971	0.006725
Zhongyun River (2020)	BPS	0.000145	0.00017
Zhujiang River (2015)	BPA	0.059171	0.222362
Zhujiang River (2015)	BPF	0.000357	0.001006
Zhujiang River (2015)	BPS	0.001264	0.002926
